# Analysis of truck occupant injury severities in fatal crashes involving trucks under different light conditions on rural roadways

**DOI:** 10.3389/fpubh.2026.1859214

**Published:** 2026-08-14

**Authors:** Yalong Yuan, Shuwei Zhang

**Affiliations:** School of Transportation, East China JiaoTong University, Nanchang, China

**Keywords:** generalized ordered logit model, light conditions, rural roadways, truck occupant injury severity, truck-involved fatal crash

## Abstract

**Introduction:**

Truck-involved fatal crashes on rural roadways often result in severe safety consequences for truck occupants, and the effects of contributing factors may vary across lighting conditions. This study aimed to examine truck occupant injury severity under daylight, dark-not-lighted, and dark-lighted conditions.

**Methods:**

Using 9,332 rural fatal truck-involved crashes from the 2012–2016 Trucks Involved in Fatal Accidents/Crashes database, we defined the dependent variable as the maximum injury severity among truck occupants, grouped into no injury, minor injury, and major injury based on the KABCO scale. Generalized ordered logit models were estimated separately for the three lighting conditions, and likelihood-ratio tests were used to justify the separate model structure.

**Results:**

The generalized ordered logit models showed better relative in-sample fit than the ordered logit models, with pseudo R-squared values of 0.19, 0.24, and 0.44 for the daylight, dark-not-lighted, and dark-lighted subsets, respectively. Driver-behavior factors – including fatigued driving, careless or inattentive driving, drug or alcohol involvement, and failure to keep in the proper lane – were associated with increased probabilities of major truck-occupant injury. Roadway-context variables, such as roadway grade, two-lane segments, adverse weather, and segments with higher posted speed limits, showed condition-specific associations with injury severity, particularly under nighttime conditions.

**Discussion:**

The findings suggest that rural lighting improvement and nighttime truck safety management should be prioritized for high-risk roadway and driver-behavior scenarios. These results underscore the need for targeted countermeasures that account for different lighting environments to mitigate severe injuries in rural truck crashes.

## 1 Introduction

Trucks (vehicles with a gross weight rating greater than 10,000 pounds) are vital to freight logistics and economic wellbeing, but they are overrepresented in terms of both the number of fatal crashes and the amount of property damage compared to passenger cars (1). In particular, truck occupants (drivers and passengers in the truck) are as much as 7 times more likely to die and 2.5 times more likely to suffer an injury than average workers (2). In truck-involved fatal crashes, which include at least one fatality, truck occupants are more likely to be injured or even killed, though truck drivers are mainly responsible for fewer of these crashes (3). During the 5-year period of 2006–2010, there were an average of 3,227 fatal crashes involving large trucks in the United States and an average of 3,736 people killed in those crashes, which caused a total of 2,081 truck occupants to die, accounting for 11.13% of the total fatalities. This means that there are at least 11 truck occupants killed for every 100 truck-involved fatal crashes. Due to the higher fatality rates of truck occupants in these crashes, research is urgently needed to better understand the key contributing factors related to truck occupant injury severities in truck-involved fatal crashes, and rational injury mitigation and prevention countermeasures should be developed to protect truck occupant safety in such crashes. In addition, truck-involved fatal crashes often involve a large number of casualties and property losses, imposing substantial social and economic burdens on truck occupants, other road users, and society (4). Therefore, the reduction of casualties in truck-involved fatal crashes plays an important role in ensuring travelers' healthy, happy, fair, and sustainable traveling. Furthermore, mitigation and prevention strategies for such fatal crashes can not only protect truck occupants in serious crashes but also effectively alleviate truck occupant injury severity in lower-injury-severity crashes.

Moreover, for truck-involved fatal crashes on rural roadways, higher crash and fatality rates were noticed from the Trucks Involved in Fatal Crashes (TIFA) dataset: 10,822 crashes occurred on rural roadways from 2006–2010, accounting for almost 66.3% of the total number of truck-involved fatal crashes. Furthermore, 1,584 truck occupants were killed in crashes that occurred on rural roadways, accounting for almost 76% of all truck occupant fatalities. This means that for the same number of truck-involved fatal crashes, the number of truck occupant fatalities in rural is three times that for urban crashes. Therefore, the higher fatality rate on rural roadways has drawn many researchers' interests worldwide (5–8). Similarly, distinct crash characteristics under different light conditions were also observed, as was evidenced by the fact that in the TIFA dataset, there were a total of 2,573 crashes in dark-not-lighted conditions, but only 308 crashes occurred in dark-lighted conditions. It is clear that trucks are more easily involved in fatal crashes under dark-not-lighted conditions, but the specific mechanisms via which light conditions affect truck occupant injury severities are not clear. Therefore, the underlying reasons for truck occupant injury severities under different light conditions must urgently be explored.

Many previous studies (5, 6, 8, 9) have found that the probability of severe injury severity is increased in dark-not-lighted conditions, and they speculated that this may be caused by truck drivers' obstructed vision, shorter sight distance and reaction time at night due to the imperfect light conditions on rural roadways. Light conditions have been viewed as an important indicator variable in many crash studies, such as Lemp et al. (10), Chen and Chen (6), Zhu and Srinivasan (11), and Xie et al. (7). However, there are some limitations regarding the use of the light conditions as an indicator variable. First, we can easily identify light conditions as a significant contributing factor for crash severity. However, some questions about light conditions are still unanswered, such as where or in which situations the light conditions will have a greater effect on crash severity. Transportation facility designers and planners are most interested in what affects crash severity under the combination of different light conditions and specific roadway segments or adverse conditions. Such information could help them better plan light facilities, but light conditions as an indicator variable cannot accomplish this task. Second, the effect of light conditions on drivers, vehicles, and road-related variables would be ignored by setting light conditions as an indicator variable. However, complicated interactive effects between light conditions and various explanatory variables exist. For example, in poor light conditions, the driver's sight, psychological perception, and behavior can be affected, which might cause poor reactions in response to curves, grades, or intersections. Moreover, drivers are more likely to be fatigued under dark conditions, which would cause them to have less reaction and braking time before a collision, leading to more serious crashes. However, if light conditions are used as an indicator variable, the interactive effects of these explanatory variables cannot be considered. In addition, awareness of truck occupant safety at night has been a major concern because it is believed that driving at night is more dangerous (1, 12). Therefore, it is urgent to ensure truck occupant safety and reduce the number and severity of truck-involved crashes under dark-lighted or dark-not-lighted conditions during nighttime.

Given these fatality-concentrated features of truck-involved fatal crashes on rural roadways under different light conditions, it is of particular importance and interest to identify contributing factors that affect truck occupant injury severity under different light conditions. It should be emphasized that this study does not model crash occurrence or crash frequency. Instead, it examines truck occupant injury severity conditional on a truck-involved fatal crash having occurred. The two main purposes of the present paper are as follows. First, by analyzing the combined effects of different light conditions and various explanatory variables on truck occupant injury severity, scenarios with a higher probability of serious truck occupant injury severity were determined. These results can be used to determine where or in which situations the light conditions will have a greater effect on crash severity and help planners optimize lighting facilities on rural roadways. Second, a comparative analysis of the contributing factors affecting truck occupant injury severity under different light conditions was performed, and reasonable mitigation and management countermeasures for truck occupant injury severity under different light conditions can be developed based on our findings.

The remainder of the paper is organized as follows: first, a literature review regarding truck-involved crash studies under different area types and light conditions is presented. Next, the TIFA dataset is described, and the statistical analysis for selected variables in different light conditions is performed. Then, the conceptual model is developed, followed by an introduction of the generalized order logit model. Afterwards, the model specification tests are explained, and the results are provided and discussed. Finally, we draw conclusions, highlight managerial implications, and discuss future research directions.

## 2 Literature review

### 2.1 Studies regarding fatal crashes involving trucks

Focusing on fatal crashes involving trucks, some studies (3, 13, 14) have been performed to identify key contributing factors, driver's responsibility and root causes of crashes, and some interesting insights have been obtained. Through analyzing fatal two-vehicle crash involving trucks and interviewing truck drivers, Helinä and Summala (3) found that other road users were principally responsible for 83% of the fatal crashes, and truck driver age, driving during evening hours and other driver fatigue related factors were identified as significant contributing factors. In addition, Brodie et al. (13) conducted a descriptive study of heavy vehicle driver fatalities by analyzing coroner's death investigation files, and the nature and extent of truck driver fatalities were found to correlate with male, solo drivers with a higher proportion of stimulants or cannabis use and lower frequency of seatbelt use. Furthermore, Zhang et al. (14) investigated truck-related fatal crashes in surface mining using 12 crash records with 13 fatalities, and improper pre-operational check and poor maintenance of trucks were indicated as two most common root causes of these crashes. More recent large-truck safety studies have increasingly adopted advanced econometric models to capture temporal instability and unobserved heterogeneity. Biglari et al. (15) re-examined large-truck at-fault crashes on rural and urban roadways in Alabama and showed that crash factors may vary over time. Habib et al. (16) analyzed the impact of roadway terrain on large-truck driver injury severity using random parameters with heterogeneity in means and variances, while Islam (17) compared fatigue- and non-fatigue-related large-truck crashes and found distinct injury-severity mechanisms across the two driver-behavior groups. Habib et al. (18) further highlighted the role of solar glare and unobserved heterogeneity in single-vehicle truck crashes on rural roads. These studies indicate that large-truck injury severity is affected by driver behavior, roadway terrain, temporal context, and visibility-related factors; however, most of them focus on crash-level or driver-level injury severity rather than maximum truck-occupant injury severity in fatal crashes under different lighting conditions.

Previous studies (1, 10, 11, 19) have explored the contributing factors that affect truck-involved crash injury severity from the crash level, which means the most serious injury severity of the occupants in the crash. From the crash-level perspective, it is possible to comprehensively analyze the impact of all participants in the crash, and some interesting, valuable, and practical results could be obtained to mitigate and manage the overall crash severity. However, truck manufacturers and safety managers are more concerned about the casualties of truck occupants in crashes, but the information about truck occupants may not be fully considered in crash-level studies. In addition, crashes with the same crash-level injury severity are assumed to have the same occupant injury severity and property damage overall in the studies. Since the heterogeneity of crashes with the same crash-level injury severity is neglected in crash-level studies (11, 20), researchers are hindered from analyzing these crashes at a deeper level, and some of the important information hidden in such crashes cannot be extracted easily. Particularly, truck-involved fatal crashes usually contain more than one casualty and larger property losses. Although these crashes have the same injury severity at the crash level, many differences in truck occupant injury severity still exist, which could be used by truck manufacturers and safety management departments to protect truck occupants from such serious crashes. Furthermore, many researchers have also studied the injury severity of truck drivers (5, 7, 21–23) and occupants (11, 24–26). These studies mainly use datasets with information about the non-fatal truck-involved crashes in a certain area, and contributing factors such as environment-, roadway-, vehicle-, driver-, and crash-related variables for driver or occupant injury severity have been identified. The results obtained from these studies have a certain role in ensuring the safety of truck drivers and occupants. Although these studies also analyzed fatal crashes to some extent, studies regarding truck occupant injury severity at serious crash levels, such as truck-involved fatal crashes, are virtually absent. To reduce the casualties of truck occupants in such serious crashes, complementary research about truck occupants in fatal crashes is performed in the present paper, and recommendations can then be given to truck manufacturers and safety management departments.

### 2.2 Spatial and temporal stability of contributing factors

Recent studies on the spatial and temporal stability of crash severity factors have primarily focused on parameter transferability across time periods, roadway contexts, and crash types. In the large-truck safety domain, Behnood and Mannering (27) examined large-truck crashes in Los Angeles and found that the effects of injury-severity factors varied by time of day and from year to year, confirming the importance of temporal instability in large-truck crash-severity modeling. Biglari et al. (15) also reported temporal instability in large-truck at-fault crash factors on rural and urban roadways in Alabama. In addition, Abdi and O'Hern (28) investigated single-vehicle truck crashes under different crash types using mixed logit models and reported significant heterogeneity across rollover, fixed-object, and run-off-road crashes. Habib et al. (29) identified both temporally stable and newly emerging factors in large-truck crashes using mixed logit models, whereas Habib et al. (16) further showed that roadway terrain can influence large-truck driver injury severity through spatiotemporally heterogeneous effects. These studies demonstrate that the determinants of large-truck crash severity are not necessarily stable across time, roadway context, or crash configuration. Nevertheless, very few studies have explicitly examined the stability and condition-specific effects of factors influencing truck occupant injury severity in fatal crashes, particularly under different lighting conditions on rural roadways.

### 2.3 Studies of crashes on rural roadways

Compared with urban roadways, rural roadways have longer mileage, relatively monotonous surroundings and relatively poor lighting facilities, all of which impact drivers' perception, reaction, and vehicle operation (5–8). Using a rural roadway crash dataset, many interesting and practical results regarding crash severity were obtained to strengthen safety management on rural roadways. In these studies, the main differences in the factors contributing to truck driver injury severity between rural and urban roadways were identified by Khorashadi et al. (5), and some risk factors, such as intersection areas, time periods, and driver drinking, were found to be significant on only rural roadways, not urban roadways. Chen and Chen (6) analyzed truck-involved crashes on rural roadways and identified individual key risk factors for single-vehicle and multi-vehicle crashes; they found that some explanatory variables, such as age, improper lane usage and failing to reduce speed, were significant for only single-vehicle crashes and not for multi-vehicle crashes. Furthermore, crashes involving single vehicles on rural roadways were analyzed by Xie et al. (7), and some contributing factors, such as age, driver drinking, light conditions and posted speed limit, were significantly (at the 0.05 level) associated with driver injury severity. In addition, Chen et al. (8) studied driver injury severity in rural interstate highway crashes and indicated that some contributing factors, such as single-vehicle crashes and drivers who were female, older adults, or driving under the influence of drugs or alcohol, significantly increase the probability of severe driver injury. Moreover, Chen et al. (9) explored the effects of different surface conditions on rural roadways on crash severity, and they found that compared with wet, icy or adverse surface conditions, dry and fine surface conditions decrease the probability of serious crashes. In summary, the probability of crashes on rural roadways is higher than that on non-rural roadways, and research regarding truck safety on rural roadways is still essential. Recent work by Yasanthi et al. (30) specifically developed crash modification factors for cold-region rural highways and found that poor visibility increases truck-related injury crashes by 50% on two-lane rural roads. Similarly, Gaweesh et al. (31) proposed a latent class-random parameter framework for large trucks on rural interstates, highlighting that unobserved heterogeneity is particularly pronounced on rural alignments. Habib et al. (18) examined single-vehicle truck crashes on rural roads and showed that solar glare and unobserved heterogeneity can affect injury-severity outcomes. In view of the relatively poor light conditions on rural roadways at night, it is urgent to determine the key risk factors affecting truck occupant injury severity by considering the characteristics of rural roadways and light conditions.

### 2.4 Research methods for light conditions in crash studies

There are two main methods to determine the effect of light conditions on crash severity. In the first method, the light conditions are viewed as an indicator variable, and the corresponding discrete choice model is built to evaluate its significance and margin effect on the crash severity. In these studies, Lemp et al. (10) identified key factors contributing to the severity of large truck crashes and found that all non-bright conditions were estimated to increase the probability of occupant fatality in the crash; moreover, they indicated that the main cause for this may be that an appropriate response is difficult to achieve for truck drivers under poorer light conditions. Similarly, Chen and Chen (6) treated light conditions as an indicator variable in their study and noted that the probability of severe injury was higher in darkness conditions for multivehicle-involved crashes. By combining the time of day and light conditions, as well as four indicator variables (daylight at peak hours, daylight off peak hours, dark-not-lighted, and dark-lighted), Zhu and Srinivasan (11) found that more severe crashes happened during “dark-but-lighted” conditions (7:30 p.m. to 5:30 a.m.) compared with the daylight and dark periods. Xie et al. (7) also studied driver injury severity on rural roadways and selected daytime and darkness as indicator variables to represent different light conditions; they indicated that darkness could in fact increase the probability of drivers being involved in non-injury crashes, which may be caused by drivers' more cautious driving and operating under darkness conditions. Moreover, Pahukula et al. (22) analyzed the main difference in contributing factors for truck-involved crashes at four time periods and categorized light conditions as dark, dawn, dusk, and other light conditions. They found that different combinations of time periods and light conditions had distinct effects on driver injury severity and that these differences were significant. Summing up, the first method for considering light conditions has some advantages, such as that explanatory variables are treated in a simple manner and the results are easy to understand. However, this method also has some limitations, such as the fact that the complex interactions between risk factors cannot be considered, which hinders researchers from evaluating and analyzing the combined effect of light conditions and various risk factors on crash severity. Therefore, safety managers are still uninformed about where and under what conditions improving light conditions can effectively mitigate crash severity.

Although the present study focuses on large-truck fatal crashes, recent visibility-related crash-severity studies also provide useful methodological and contextual references. Ullah et al. (32) examined the effects of sight distance, road conditions, and weather on intersection crash severity using a random parameters logit approach with heterogeneity in means and variances, showing that visibility and roadway-environmental factors should be considered jointly when interpreting crash severity. Ullah et al. (33) applied ordinal probit modeling with bootstrap validation to injury-severity risks at visually obstructed intersections, further emphasizing the need to consider sight obstruction and model validation in ordinal injury-severity analysis. These studies support treating lighting conditions as part of a broader visibility and roadway-context problem rather than merely as a simple categorical indicator.

To consider the combined effect of light conditions and various risk factors on crash severity more comprehensively, the second method divides the overall dataset into subsets by considering different light conditions. Then, corresponding separated estimation models are built according to different light conditions data subsets, and a comparative analysis is performed to show the distinct effects of various factors under different light conditions. In these studies, Anarkooli and Hosseinlou (12) divided the overall dataset into daylight, dark, and dark with street light data subsets and analyzed contributing factors affecting crash severity on rural two-lane roadways under different light conditions. Uddin and Huynh (1) divided the overall dataset into six subsets according to the combination of light conditions and land area types and developed separate injury-severity models for truck-involved crashes. Al-Bdairi et al. (34) focused more specifically on large trucks by developing separate mixed logit models for run-off-road crashes under lighted and dark conditions, and their results indicated that the effects of driver, traffic-flow, geometric, land-use, and time-related factors can differ substantially between lighting conditions. Alogaili and Mannering (35) also applied a day-night split to pedestrian injury severity while accounting for temporal instability and unobserved effects. To sum up, the second method has advantages that overcome the limitations of treating light conditions as a single indicator. In this manner, the combined effects of different light conditions and various explanatory variables on crash severity can be determined, and the results can help identify key factors under specific light conditions for mitigating truck-involved fatal crashes on rural roadways.

In summary, recent studies have substantially advanced the understanding of large-truck crash severity, temporal instability, unobserved heterogeneity, rural roadway effects, and lighting-related differences. However, most existing work has concentrated on crash-level severity, driver injury severity, or specific crash types. Studies that define the dependent variable as the maximum injury severity among truck occupants in fatal truck-involved crashes remain limited. In addition, few studies have compared how driver-, vehicle-, roadway-, environmental-, and crash-related factors affect truck-occupant injury severity across daylight, dark-not-lighted, and dark-lighted rural roadway conditions. Elucidating these condition-specific effects can help transportation agencies and truck safety managers identify where lighting improvement, nighttime enforcement, speed management, and occupant-protection strategies are most needed.

## 3 Data preparation

In the present study, data regarding truck-involved fatal crashes from 2012 to 2016 were retrieved from the TIFA database. The TIFA files contain records of all fatal crashes involving medium and heavy trucks in all 50 states and the District of Columbia by years, and each record includes at least one fatality in the crash. The associated crash variables and vehicle variables related to the truck and occupant variables are available through virtual links to the Fatality Analysis Reporting System (FARS) dataset, which is recognized as the most reliable national database of this type. Since comprehensive risk factors to truck occupant injury severity under different light conditions are the main interest of this paper, only crash records including environmental, roadway, vehicle, driver, and crash characteristics of interest were extracted. After data screening, preliminary sorting, and eliminating default data cases, we obtained 14,007 valid case records from the 16,135 total records. To identify contributing factors to truck occupant injury severity under different light conditions on rural roadways, all truck-involved fatal crash that occurred on rural roadways were extracted from our dataset. Subsequently, 9,332 valid rural roadways crash records were divided into a daylight subset (6,451), dark-not-lighted subset (2,573), and dark-lighted subset (308).

Because the present study focuses exclusively on truck-involved fatal crashes recorded in the TIFA/FARS system, each crash included in the analysis involved at least one fatality at the crash level. Therefore, the “no injury” category in this study refers to no injury to truck occupants within fatal crashes, rather than property-damage-only crashes at the crash level. Accordingly, the conventional underreporting issue commonly associated with less severe or property-damage-only police-reported crashes is not directly applicable to the fatal-crash-only sample used in this study. Nevertheless, the findings of this study should be interpreted within the scope of fatal truck-involved crashes and should not be directly generalized to non-fatal or property-damage-only truck crashes.

The dependent variable was defined as the maximum injury severity among the truck driver and passengers. This definition was adopted because the objective of this study is to evaluate the most serious safety consequence to occupants inside the truck unit, rather than driver injury alone. This aggregation is useful for identifying severe occupant-protection scenarios and developing truck safety management implications. However, this definition does not distinguish whether the most severely injured occupant was the driver or a passenger. Therefore, the effects of driver-behavior variables should be interpreted as associations with the maximum truck-occupant injury outcome, and driver-only injury severity may produce different parameter estimates.

The truck occupant injury severity is identified according to the highest injury severity among the truck driver and passengers in the crash. The KABCO injury scale is developed by the National Safety Council (NSC) is used in the TIFA database to define the injury severity of truck occupants: K is a fatal injury, which results in death of occupants within 30 days of the crash; A is an incapacitating injury, which is described as a disabling injury due to which the injured cannot leave the scene without assistance (i.e., broken bones, severe cuts, unconsciousness, etc.); B is a non-incapacitating injury, which is described as a non-disabling injury but visible injury (i.e., minor cuts, swelling, limping, bruises and abrasions, etc.); C is a possible injury; and O represents no apparent injury in the KABCO coding system. To obtain a reasonable and fine estimation result, it is essential and basic to have a sufficient sample size for each occupant injury severity. However, due to the lower proportion of A-injured occupants (4.9%) and the higher proportion of O-injured occupants (56.5%), this may lead to inaccurate and biased estimation results. Referring to the method used in similar studies (6, 19, 21, 22), the KABCO injury scale codes in the TIFA dataset are consolidated into three levels: major injury (KA), which includes incapacitating and fatal injuries; minor injury (BC), which includes non-incapacitating and possible injuries; and no injury (O).

To quantitatively identify the influences of the categorical and nominal variables in truck occupant injury severity, we set dummy variables in the crash-, environmental-, roadway-, vehicle-, and driver-related categorical variables. They are transformed into binary variables, and the coding of each category is reported in Table 1. Posted speed limit was treated as a roadway-context variable rather than a direct proxy for actual vehicle operating speed. Actual pre-crash operating speed was not available in the TIFA/FARS data. Therefore, the estimated effects of speed-limit variables should be interpreted as associations between posted speed-limit environments and truck occupant injury severity, rather than causal effects of actual travel speed. Table 1 indicates that crashes that occurred under daylight conditions accounted for 69% of the total number of crashes. Among them, the proportion of major injury among truck occupants was lower (18.8%), and the rate of single-vehicle involved crashes was the lowest (12.2%). Regarding truck-related characteristics in crashes, trucks weighing more than 33,000 pounds (74.7%) and trailers (64.7%) had the highest proportion of crashes under this condition. For truck drivers under daylighted conditions, the proportion of young (6.0%) and older adults drivers (12.4%) was the highest in all conditions.

**Table 1:** Statistical-descriptive analysis of the truck-involved fatal crash (*N* = 9,332).

Variable	Categories	Overall sample	Daylight	Dark-not-lighted	Dark-lighted
		(*N* = 9,332)	*N* = 6,451	*N* = 2,573	*N* = 308
The crash-related variables					
Occupant injury severity in truck	Fatal injury (K)	14.5%	13.5%	17.3%	10.1%
	Incapacitating injury (A)	4.9%	5.3%	3.9%	2.9%
	Non-incapacitating injury (B)	12.6%	13.3%	11.2%	9.1%
	Possible injury (C)	11.6%	12.1%	10.3%	11.7%
	No injury (O)	56.5%	55.7%	57.3%	66.2%
Manner of collision	Front-to-rear: 1, others: 0	17.5%	16.1%	20.2%	19.5%
	Front-to-front: 1, others: 0	19.5%	19.7%	20.0%	11.7%
	Angle-front-to-side: 1, others: 0	17.5%	18.6%	13.0%	31.2%
Number of vehicles involved	Single vehicle: 1, others: 0	15.3%	12.2%	19.2%	13.6%
The environment and roadway-related variables					
Light condition	Daytime: 1, others: 0	69.1%	100%	0%	0%
	Dark-not-lighted: 1, others: 0	27.3%	0%	100%	0%
	Dark -lighted: 1, others: 0	3.3%	0%	0%	100%
Atmospheric condition	Rain, snow etc.: 1, Clear: 0	17.2%	16.4%	19.8%	11.7%
Surface condition	Wet, ice etc.: 1, Dry: 0	18.5%	17.6%	20.8%	15.9%
Day of week	Weekday: 1, others: 0	84.5%	87.3%	77.9%	80.5%
Roadway alignment	Curve: 1, Straight: 0	19.1%	20.1%	17.5%	10.1%
Roadway grade	Grade etc.: 1, Level: 0	29.4%	30.0%	29.0%	18.8%
Surface type	Blacktop: 1, others (concrete, etc.): 0	90.0%	90.6%	89.2%	83.8%
Number of lanes per direction	≤2 lanes: 1, others: 0	83.6%	85.2%	81.1%	71.4%
Posted speed limit	≤40 mile/h: 1, others: 0	5.5%	6.2%	3.0%	11.4%
	45–55 mile/h: 1, others: 0	50.8%	55.0%	40.1%	51.6%
	60–70 mile/h: 1, others: 0	39.7%	35.2%	51.7%	34.1%
	>70 mile/h: 1, others: 0	4.0%	3.6%	5.2%	2.9%
The vehicle-related variables					
Body type	Truck-tractor: 1, single unit truck: 0	65.6%	64.7%	67.4%	68.5%
Truck weight	≥33,001 lbs.: 1; 10,001–33,000 lbs.: 0	76.0%	74.7%	79.2%	76.0%
Vehicle age	≤5 years: 1, others: 0	43.7%	41.5%	49.2%	45.5%
	>5 and ≤ 10 years: 1, others: 0	30.8%	30.2%	31.9%	35.7%
	>10 years: 1, others: 0	25.5%	28.4%	18.9%	18.8%
Restraint system	Shoulder belt not used: 1; others: 0	17.6%	18.0%	17.0%	14.6%
The driver-related variables					
Gender	Male: 1	97.00%	96.90%	97.30%	95.80%
	Female: 0	3.00%	3.10%	2.70%	4.20%
Age	Young driver: 1, others: 0	5.70%	6.00%	5.10%	5.20%
	Middle-aged driver: 1, others: 0	82.00%	81.60%	82.90%	84.70%
	Older driver: 1, others: 0	12.30%	12.40%	12.00%	10.10%
License	Valid = Yes: 1, others: 0	96.90%	96.90%	97.20%	95.80%
History of crashes	Crashes = Yes: 1, others: 0	14.30%	14.40%	13.60%	17.50%
History of convictions	Suspensions = Yes: 1, others: 0	7.00%	6.80%	7.40%	8.10%
	Speeding = Yes: 1, others: 0	24.10%	23.50%	25.50%	25.00%
	Other convictions = Yes: 1, others: 0	22.50%	21.90%	24.20%	21.40%
Driver drowsy or fatigued	Drowsy = Yes: 1, others: 0	1.20%	1.50%	2.10%	1.30%
Influence of drugs	Drug = Yes: 1, others: 0	1.30%	1.00%	2.10%	2.30%
Driver careless/inattentive	Careless = Yes: 1, others: 0	4.40%	4.80%	3.70%	2.90%
Driver drinking	Yes: 1, others: 0	2.60%	1.60%	4.90%	3.90%
Failure to yield right-of-way	Yes: 1, others: 0	3.40%	2.70%	2.60%	5.20%
Failure to keep in proper lane	Yes: 1, others: 0	6.20%	7.50%	7.40%	3.90%

In addition, crashes that occurred under dark-not-lighted conditions accounted for 28% of the total number of crashes. Among them, truck occupants accounted for the highest proportion of those who suffered major injuries (21.2%), and the rates of single-vehicle (19.2%), front-to-front (20.0%) and front-to-rear (20.2%) crashes were higher. Additionally, most trucks involved in the crashes weighed more than 33,000 pounds (79.2%) and had a vehicle age of less than 5 years (49.2%). The proportions of drivers who were middle-aged (82.9%), drinking (2.1%) and under influence of the drugs (4.9%) were higher, and it should be noted that the proportions of drivers who were driving carelessly (3.7%) and fatigued (2.1%) were the highest under this condition. In the case of unfavorable weather, such as snow and rain (19.8%), adverse road surface conditions (20.8%), and roadway segments with posted speed limits greater than 60 miles/h (56.9%), the proportions of crashes were the highest under dark-not-lighted conditions among the three lighting conditions.

Finally, crashes that occurred under dark-lighted conditions accounted for 3% of the total number of crashes. This descriptive difference suggests that the distribution of fatal truck-involved crashes varied substantially across lighting conditions, but it should not be interpreted as exposure-normalized crash risk. Among them, truck occupants had the lowest proportion of suffering major injuries (13.0%), and the rates of single-vehicle (13.6%) and front-to-front (11.7%) crashes were lower. For trucks involved in these crashes, most weighed more than 33,000 pounds (76.0%) and had a trailer (68.5%) body type. Furthermore, drivers under these conditions had the following characteristics: the highest proportion of middle-aged drivers (84.7%), higher proportions of driving under the influence of drugs (3.9%) and alcohol (2.3%), and the lowest proportions of careless (2.9%) and fatigued (1.3%) driving. Compared with the dark-not-lighted subset, the dark-lighted subset showed lower proportions of crashes under unfavorable weather (11.7%), adverse road surface conditions (15.9%), and roadway segments with posted speed limits greater than 60 miles/h (37%). However, these descriptive comparisons are based on crash counts and should not be interpreted as exposure-normalized crash risk.

## 4 Methodology

### 4.1 Conceptual framework of the truck occupant injury severities model

**Figure 1:**
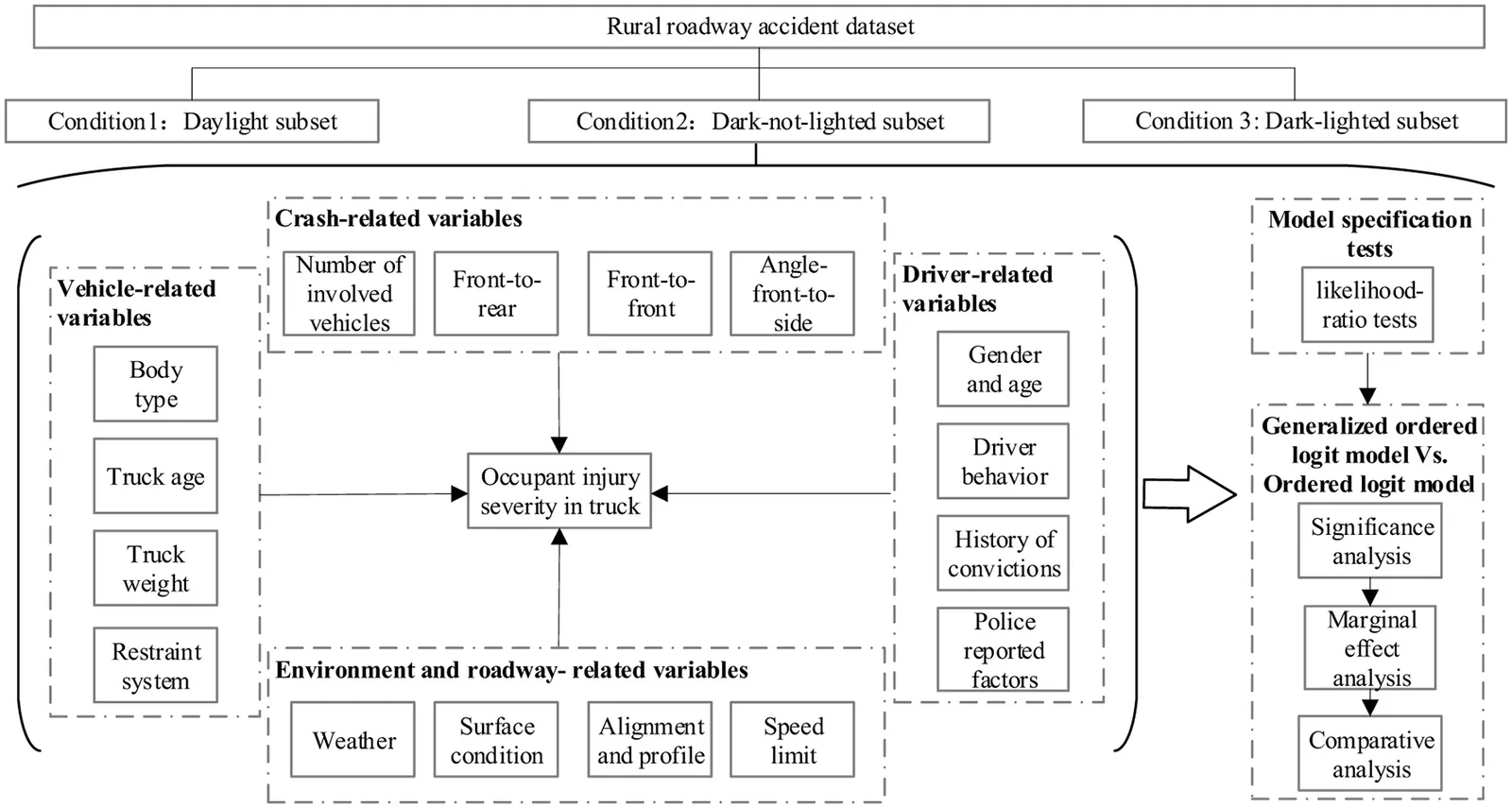
Conceptual framework of the analysis of truck occupant injury severities under different light conditions on rural roadways.

The conceptual framework of the truck occupant injury severities analysis under different light conditions on rural roadways was constructed, as shown in Figure 1. First, all crashes occurring on rural roadways were extracted from the TIFA database, and a data subset mainly containing rural truck-involved crashes was established. Then, the overall rural dataset was divided into three data subsets according to different light conditions: a daylight subset, dark-not-lighted subset, and dark-lighted subset. From the driver-, vehicle-, crash-, environment-, and roadway-levels, corresponding explanatory variables (shown in rectangular boxes) were selected from the dataset, and the key contributing factors to truck occupant injury severity were mainly identified from these factors. Before three separate models according to different light conditions were developed, likelihood ratio tests were performed to certify the statistical validity for three separate models in comparison with the overall sample model. Then, the generalized ordered logit model and ordered logit model were used to fit all light conditions datasets individually, and a more appropriate model was chosen according to certain fitness parameters in the subsequent study. Furthermore, the key factors contributing to injury severity under different light conditions were identified by a statistical significance test, and their marginal effects were calculated to simulate their dynamic effects on occupant injury severity. Finally, the similar and different effects of the explanatory variables under different light conditions were summarized via a comparative analysis, and thus corresponding recommendations to safety planners and managers could be given.

### 4.2 Generalized ordered logit model

In this study, the truck occupant injury severities are scaled into three levels: no injury (O), minor injury (BC), and major injury (KA). The higher injury severity level means more serious casualties of truck occupants in the crash. Let $Y_{i}^{*}$ be the latent continuous variable of the discrete truck occupant injury severity level $Y_{i}^{*}$ in crash *i*; $Y_{i}^{*}$ can be estimated as Equation 1 follows:


$$\begin{array}{c} Y_{i}^{*}=X_{i}^{\prime }\beta +\varepsilon \end{array}$$ (1)

where *X*_*i*_ is a *p* × 1 vector of explanatory variables, β is a *p* × 1 vector of regression coefficients for *X*_*i*_, ε is a *p* × 1 vector of error term capturing the effect of unobserved factors not included in the *X*_*i*_, and *p* is the number of explanatory variables.

From a methodological perspective, discrete choice models are widely used in safety studies because of their concise form, fine logical structure and better fit to crash datasets (36–38). Among them, multinomial logit, ordered logit, ordered probit, and mixed logit models are the most practical and prominent types of discrete choice models, which have been applied by a large number of scholars in safety studies (19, 39–42). For ordinal dependent variables such as crash severity, Williams (43), Wang and Abdel-Aty (44), and Kaplan and Prato (45) recommended that an ordered response model may be a better choice for modeling such variables. In such models, the ordinal nature of independent variables could be fully considered and used to obtain more parsimonious results, and incorrect, incomplete, or misleading estimated results can be avoided. Among them, the ordered logit model is widely used in transportation safety research because of its concise form and fine ordinal responsiveness (19, 42). In addition, Ye and Lord (46) indicated that an ordered response model could minimize the bias produced by an insufficient sample size. In present paper, the sample size for dark-lighted conditions is smaller, and the adoption of the ordered logit model can ensure the performance of the injury severity models for small sample sizes. In this paper, the truck occupant injury severity is taken as a discrete ordered response dependent variable, and the ordered logit model can be written in terms of the probability of the *j*-th injury severity for a given crash *i* as Equation 2 follows (43):


$$\begin{array}{c} P\left(Y_{i}>j\right)=P_{ij}=\frac{\exp\left(\alpha_{j}+X_{i}^{\prime }\beta \right)}{1+\exp\left(\alpha_{j}+X_{i}^{\prime }\beta \right)}\text{, }where\;j=1,2,\ldots ,J-1 \end{array}$$ (2)

where *P*_*ij*_ is a probability of *j*-th injury severity for a given crash *i*, β is a *p* × 1 vector of parameters to be estimated, α_*j*_ is a *p* × 1 vector of cut-off points for the *j*-th cumulative logit; *j* is the crash severity level (level 1 = No injury; level 2 = minor injury; level 3 = major injury), and *J* is the number of severity levels (in this study *J* = 3). An important assumption, which is also well known as the parallel line assumption or proportional odds assumption, is associated with the ordered logit model and demands that the relationship between any two injury categories in the dependent variable group is the same; therefore, the β values for all *j*-th severity levels will be the same (43, 44).

However, in practical applications, Wang and Abdel-Aty (44), Kaplan and Prato (45), Mooradian et al. (47), and Sasidharan and Menéndez (48) found that the parallel line assumption is too restrictive and is often violated by some explanatory variables when there are too many variables in the analysis. The violation of such an assumption could lead to the formulation of incorrect or misleading estimated results (49). The generalized ordered logit model (50), which is also known as the partial proportional odds model, is recommended as a better alternative model for ordinal response data. By relaxing the proportional odds assumptions for some or all explanatory variables, the generalized ordered logit model can obtain more correct and complete results than the ordered logit model (45). For a more parsimonious and easy-to-understand layout (43), a gamma parameterization of the generalized ordered logit model can be specified in terms of the probability of the *j*-th injury severity for a given crash *i* as Equation 3 follows (50):


$$\begin{array}{c} P\left(Y_{i}>j\right)=P_{ij}=\frac{\exp\left[\alpha_{j}+\left(X_{i}^{\prime }\beta_{j}+X_{2i}^{\prime }\gamma_{j}\right)\text{,}\right]}{1\text{+}\exp\left[\alpha_{j}+\left(X_{i}^{\prime }\beta_{j}+X_{2i}^{\prime }\gamma_{j}\right)\right]}\\ where\;j=1,2,\ldots ,J-1 \end{array}$$ (3)

where γ_*j*_ is a *p* × 1 vector of estimated parameters that does violate the proportional odds assumption associated with a subset of observed explanatory variables *X*_2*i*_ and β_*j*_ is a *p* × 1 vector of estimated parameters that are the same for all values of the *j*-th injury severity. In this model, each independent variable has one β coefficient and *J* – 2 γ coefficients, and the specific calculation method for the γ coefficients was introduced in detail by Williams (43). We can better judge whether the parallel line assumptions of explanatory variables are being violated by examining the γ coefficients, and the assumption is met for a variable when the γ coefficients for the variable all equal zero. In the three-level injury-severity setting, a significant gamma coefficient indicates that the effect of a predictor is not constant across the two cumulative severity transitions: from no injury to any injury, and from minor-or-worse injury to major injury. Therefore, the gamma coefficient helps identify variables whose effects differ between lower-severity and higher-severity transitions. This flexibility is the main advantage of the generalized ordered logit model over the standard ordered logit model. From the above, the probabilities that *Y*_*i*_ will take on each of the values *1, …, J* can be determined as Equation 4–6 follows:


$$\begin{array}{c} P\left(Y_{i}=1\right)=1-P_{i1} \end{array}$$ (4)

$$\begin{array}{c} P\left(Y_{i}=j\right)=P_{i,j-1}-P_{ij}\;\text{Where }j=2,\ldots ,J-1 \end{array}$$ (5)

$$\begin{array}{c} P\left(Y_{i}=\text{J}\right)=P_{i,J-1} \end{array}$$ (6)

In general, we can obtain the sign and significance of explanatory variable from the parameter estimation results of the generalized ordered logit model, but it is difficult to obtain a tangible feel for the magnitudes and importance of the effects of these explanatory variables on the injury severity. In addition, it must be noted that the intermediate categories' coefficients should be interpreted with caution, since the sign of the estimated parameter does not always determine the direction of the effect of the intermediate severity outcomes (43, 51). To more accurately and tangibly understand the probability change of crash severity when certain explanatory variable increase or decrease one percent while all others remain equal, Train (52), Xie et al. (7), Anarkooli and Hosseinlou (12), and Uddin and Huynh (1) recommend that the direct-marginal and cross-marginal effects of explanatory variables can be calculated using partial derivatives of various explanatory variable in the crash severity function, which are formulated as Equations 7, 8. Among them, a direct-marginal effect represents the change in the probability of injury severity *j* in crash *i* given a change in explanatory variable *k*, and a cross-marginal effect indicates how a change in an explanatory variable *k* of injury severity *j* changes the probabilities for all other injury severity by the same percent for crash *i* (52).


$$\begin{array}{c} Direct\;E_{X_{ijk}}^{P_{ij}}\text{=}\frac{\partial P_{ij}}{\partial X_{ijk}}\text{=}\beta_{k}P_{ij}\left(1-P_{ij}\right) \end{array}$$ (7)

$$\begin{array}{c} Cross\;E_{X_{ijk}}^{P_{ij}}\text{=}\frac{\partial P_{i\text{m}}}{\partial X_{ijk}}=-\beta_{k}P_{im}P_{ij} \end{array}$$ (8)

where *X*_*ijk*_ is the independent variable *k* associated *j*-th injury severity for crash *i*; $DirectE_{X_{ijk}}^{P_{ij}}$represents direct-marginal effect, which means the impact of a change in independent variable *X*_*ijk*_ on the *P*_*ij*_ to be in *j*-th injury severity, and $CrossE_{X_{ijk}}^{P_{i\text{m}}}$ represents cross-marginal effect, which describes the impact of a change in independent variable [[Inline Image]] of *m*-th injury severity (*m* ≠ *j*) on the *P*_*i*m_ for crash *i*.

### 4.3 Model specification tests

Likelihood ratio tests have been adopted by many researchers (1, 53) to check the suitability of separate models over the full model. In the present study, similar tests were also performed to check the statistical justification of the three developed models under different light conditions. Specifically, the tests were performed to check the significance of the following:

(1) The full model for all trucks involved in fatal crashes in rural areas compared with the three separate models under daylight, dark-not-lighted, and dark-lighted conditions.
(2) Three separate models under daylight, dark-not-lighted, and dark-lighted conditions compared individually with each other.

The first log likelihood ratio test for the transferability of the coefficient from the full model to three separate models is as Equation 9 follows:


$$\begin{array}{c} LR_{all}=-\text{2}[\mathrm{LL}(\beta_{all})-\mathrm{LL}(\beta_{Daylight})-\mathrm{LL}(\beta_{Darknotlighted})\\ -\mathrm{LL}(\beta_{Darklighted})] \end{array}$$ (9)

where LL(β_*all*_) is the log-likelihood at convergence of the overall model (−7,378.41); LL(β_*Daylight*_) is the log-likelihood at convergence of the daylighted model (−5,151.00); LL(β_*Darknotlighted*_) is the log-likelihood at convergence of the dark-not-lighted model (−1,907.69); LL(β_*Darklighted*_) is the log-likelihood at convergence of the dark- lighted model (−210.41); and *LR*_*all*_ is a statistic value that follows χ^2^ distribution, with the null hypothesis is that there is no statistically significant parameter difference between the full model and separate model. In present study, *LR*_*all*_= 218.62, which is greater than the critical value of the chi-square statistic with 95 degree of freedom at the 99% confidence level (129.97). Therefore, we have 99% confidence to reject the null hypothesis, and the models have significantly different model parameters.

In the second log likelihood ratio test for transferability, the coefficients for the three separate models are individually compared with each other as Equation 10 follows:


$$\begin{array}{c} LR_{k_{1}k_{2}}\text{=}-\text{2}\left[{\text{LL}}_{k_{1}k_{2}}(\beta^{k_{1}k_{2}})-{\text{LL}}_{k_{1}}(\beta^{k_{1}})\right] \end{array}$$ (10)

where ${\text{LL}}_{k_{1}k_{2}}(\beta^{k_{1}k_{2}}$ is the log-likelihood at convergence of the *k*_2_ lighting conditions model (using *k*_2_'s data) while using the data corresponding to *k*_1_'s light conditions and ${\text{LL}}_{k_{1}}(\beta^{k_{1}})$ is the log-likelihood at convergence of the model using *k*_1_'s light conditions data.

The values of *LR*_*k*_1_*k*_2__for different light conditions models are calculated, and the results of the second transferability are reported in Table 2. Each test statistic is greater than the critical value of the chi-square statistic with corresponding degree of freedom at the 99% confidence level. Therefore, the results further validate that each light conditions model is statistically justified.

**Table 2:** Results of the second transferability for different light conditions models.

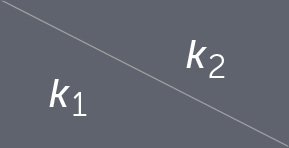	Daylight	Dark-not-lighted	Dark-lighted
Daylight	0	2,100 (*df* = 26)	1,990 (*df* = 22)
Dark-not-lighted	1,600 (*df* = 24)	0	140 (*df* = 22)
Dark-lighted	870 (*df* = 24)	920 (*df* = 26)	0
Critical value	χ^2^_(*P* = 0.01, *df* = 24)_ = 42.98	χ^2^_(*P* = 0.01, *df* = 26)_ = 45.64	χ^2^_(*P* = 0.01, *df* = 22)_ = 40.29

### 4.4 Model fitting results

In this study, generalized ordered logit model are fitted using a user-written program in Stata (43). First, Variance Inflation Factor (VIF) diagnostics are conducted for the predictor variables in each of the three lighting-condition models. The results show that all VIF values are below 6, which is well below the commonly recommended threshold of 10. This indicates that multicollinearity is not a severe concern in our study and does not substantially affect the reliability of the parameter estimates. Then, Wald tests are performed on each variable to determine whether the observed explanatory variable meets the proportional odds assumption. The variables that do not violate the proportional odds assumption are selected as constraints to impose in the final model. Finally, the maximum likelihood estimation technique is adopted to estimate the coefficients in this program, and the gamma coefficients for only these variables that are not constrained to meet the proportional odds assumption are presented (43).

For more suitable results, the ordered logit model and generalized ordered logit model for all separated models under different light conditions are fitted individually, and some fitness criteria, such as the McFadden pseudo R-squared, Akaike's information criterion (AIC) and Bayesian information criterion (BIC), which are formulated as Equations 11 – 13, are adopted for assessing which model is more appropriate. McFadden pseudo R-squared suggests the improvement level of the full model compared with the intercept model, in which larger values indicate better fitness (52). In addition, Posada and Buckley (54) recommended that both AIC and BIC can be used to compare the relative plausibility of two methods in a particular model, and smaller AIC and BIC values indicate a better-fitting result.


$$\begin{array}{c} Pseudo\;R^{2}=1-\left[\ln\;\overset{\wedge }{L}\left(M_{full}\right)/\ln\;\overset{\wedge }{L}\left(M_{\text{constant}}\right)\right] \end{array}$$ (11)

$$\begin{array}{c} AIC=-2\text{ln}\overset{\wedge }{L}\left(M_{full}\right)+2p \end{array}$$ (12)

$$\begin{array}{c} BIC\text{= }-\text{2ln}\overset{\wedge }{L}(M_{full})+p\times \text{ln(}N) \end{array}$$ (13)

where $\overset{\wedge }{L}\left(M_{full}\right)$ is the estimated likelihood of full model with constant and all predicting variables, $\overset{\wedge }{L}\left(M_{\text{cons}\tan\text{t}}\right)$ is the estimated likelihood of intercept model with constant term only, *p* is the number of estimated parameters in the model, and *N* is the number of the observations.

The fitting results of the ordered logit model and generalized ordered logit model for the different light conditions data subsets are reported in Table 3. These results show that higher pseudo R-squared values and smaller AIC and BIC values were obtained in the generalized ordered logit model in each lighting conditions model, indicating better model fitness to the dataset. However, this does not necessarily mean that the generalized ordered logit model is superior to the ordered logit model, but it does indicate that the generalized ordered logit model is more suitable for present study. Therefore, the generalized ordered logit model is adopted, and the corresponding fitting results are analyzed in a subsequent study.

**Table 3:** Fitness results of the ordered logit model and generalized ordered logit model.

Fitness criteria	Overall sample	Daylight	Dark-not-lighted	Dark-lighted
Generalized ordered logit model				
Number of observations	9,332	6,451	2,573	308
Log likelihood at zero	−11,467.61	−8,741.60	−2,970.76	−415.61
Log likelihood at convergence	−7,378.41	−5,151.00	−1,907.69	−210.41
Pseudo R-squared	0.20	0.19	0.24	0.44
BIC	14,100.81	9,723.45	4,123.56	512.74
AIC	13,769.50	9,685.35	3,923.84	458.90
Observations-to-parameters ratio (N/p)	207:1	143:1	56:1	7:1
Ordered logit model				
Number of observations	9,332	6,451	2,573	308
Log likelihood at zero	−10,845.75	−8,436.76	−3,162.97	−424.19
Log likelihood at convergence	−6,897.17	−4,835.48	−1,973.55	−216.13
Pseudo R-squared	0.16	0.17	0.18	0.23
BIC	14,132.55	9,995.52	4,237.65	644.27
AIC	13,868.33	9,744.96	4,021.1	506.25
Observations-to-parameters ratio (N/p)	311:1	143:1	56:1	7:1

Although the generalized ordered logit model showed better relative fit than the ordered logit model according to pseudo R-squared, AIC, and BIC, these indices should be interpreted as measures of in-sample relative model fit rather than out-of-sample predictive performance. This distinction is particularly important for the dark-lighted subset, which contains a much smaller number of observations than the daylight and dark-not-lighted subsets. Therefore, the relatively high pseudo R-squared of the dark-lighted model should not be interpreted as evidence of superior predictive capability. Instead, the dark-lighted model is used to provide exploratory evidence on condition-specific associations between explanatory variables and truck occupant injury severity.

Because the objective of this study is to identify and compare contributing factors under different lighting conditions rather than to develop a crash-level prediction tool, a separate holdout validation or cross-validation procedure was not incorporated in the original modeling framework. Therefore, model-fit statistics are used only to compare relative in-sample model suitability. Future research should further evaluate the predictive performance of light-condition-specific models using holdout samples, cross-validation, confusion matrices, and sensitivity/specificity measures for the major injury category.

## 5 Estimation results and discussion

The estimated results of the gamma parameterization of the generalized ordered logit model for different light conditions were obtained and are reported in Table 4. From the results, the significant factors contributing to truck occupant injury severity under different light conditions were identified via a statistical significance test. Then, the marginal effects of various explanatory variables were also evaluated and are reported in Table 5, and the sign and magnitude of the marginal effect on truck occupant injury severity were analyzed.

**Table 4:** Estimation results of gamma parameterization of generalized ordered logit model for different light conditions.

Variables	Categories	Overall sample	Daylight	Dark-not-lighted	Dark-lighted
Beta					
The crash level					
Manner of collision	Front-to-rear = yes: 1	**0.13** ^*^	**0.10** ^*^	**0.16** ^*^	**0.06**
	Front-to-front = yes: 1	0.88^**^	0.90^***^	0.89^***^	1.05^***^
	Angle - front-to-side, right angle = yes: 1	0.27^***^	0.30^***^	**−0.03**	0.56^***^
Number of vehicles involved	Single vehicle = 1	1.11^***^	1.45^***^	0.40^***^	−0.75^***^
The environment and road level					
Atmospheric condition	Rain, cloudy, snow, etc.=1	**0.08**	**0.01** ^*^	0.23^**^	0.02^**^
Day of week	Weekday = 1	0.22^***^	0.14^**^	0.27^***^	0.18^*^
Surface condition	Wet, ice, snow, etc. =1	−0.26^***^	−0.26^***^	−0.20^**^	**0.08**
Roadway alignment	Curve = 1	0.24^***^	0.19^***^	**0.38** ^*^	0.66^***^
Roadway grade	Grade, uphill, downhill, etc.= 1	**0.05**	–**0.04**	0.24^***^	−0.06^**^
Surface type	Blacktop = 1	**0.05**	**-0.03**	**0.09**	–**0.15**
Posted speed limit	≤40 mile/h = 1	−0.74^***^	−0.86^***^	−0.40^***^	−1.17^**^
	45–55 mile/h = 1	−0.10^***^	0.02^**^	−0.32^**^	–0.83^*^
	60–70 mile/h = 1	0.10^***^	0.15^**^	0.05^**^	–0.49^*^
Number of lanes per direction	≤2 lanes =1	0.13^**^	**0.03**	**0.26** ^*^	–**0.05**
The vehicle level					
Body type	Truck-tractor = 1	−0.20^***^	−0.23^***^	–**0.08**	–0.08^*^
Truck weight	33,001 lbs. or more = 1	−0.54^***^	−0.63^***^	−0.51^***^	−0.49^***^
Restraint system	Shoulder and lap belt not used = 1	1.30^***^	1.27^***^	1.18^***^	1.11^***^
Vehicle age	≤5 years = 1	–**0.01**	–0.05^*^	**0.22** ^*^	–0.22^*^
	>5 and ≤ 10 years = 1	**0.01**	–0.05^*^	0.30^**^	–**0.08**
The driver level					
Gender	Male = 1	−0.27^***^	−0.31^**^	**−0.29** ^*^	**0.06** ^*^
Age	≤25 years =1	−0.11^**^	**−0.10** ^*^	**0.05** ^*^	**0.15** ^*^
	≥65 years =1	0.23^***^	0.23^**^	0.24^**^	−0.08^**^
License class compliance	Valid = yes: 1	−0.16 ^**^	**−0.19**	−0.38^***^	−0.43^**^
Driver drinking	Yes: 1	**0.43** ^*^	0.47^**^	0.39^***^	1.16^**^
History of crashes	Yes: 1	**0.07** ^*^	**0.10**	**−0.07**	–**0.10**
History of convictions	Suspensions = yes = 1	**0.14** ^*^	**0.12**	**0.39**	**0.20**
	Speeding = yes: 1	−0.13^***^	**−0.16**	**−0.06**	**0.07**
	Driving while intoxicated = yes: 1	**−0.24** ^*^	**−0.35** ^*^	**−0.36**	–**0.02**
	Other convictions = yes: 1	**0.02** ^*^	**−0.05**	−0.03^***^	–**0.17**
Driver behavior	Drowsy or fatigued = yes: 1	1.99^***^	1.64^***^	2.57^***^	3.45^***^
	Under the influence of drugs or medication = yes: 1	0.65^***^	0.51^**^	0.63^**^	**0.04** ^**^
	Careless/inattentive = yes: 1	0.99^***^	0.87^***^	1.39^***^	0.24^***^
	Failure to keep in proper lane = yes: 1	1.10^***^	0.74^***^	0.90^***^	2.13^***^
	Failure to yield right-of-way = yes: 1	−0.74^***^	−0.76^***^	−0.71^***^	−1.35^***^
Gamma					
Number of vehicles involved	Single vehicle =1	1.33^***^	1.39^***^	1.58^***^	3.42^***^
Atmospheric condition	Rain, cloudy, snow, etc.=1	−0.15^**^	−0.33^**^	–	–
Surface type	Blacktop = 1	–	–	0.67^**^	−0.56^***^
Roadway alignment	Straight Curve = 1	–	–	–	1.82^**^
Vehicle age	≤5 years = 1	–	0.14^***^	0.58^***^	1.44^***^
	Front-to-front = yes: 1	−0.72^***^	−0.55^***^	−1.09^***^	−2.67^***^
	Angle - front-to-side, right angle = yes: 1	−0.49^**^	−0.50^***^	−0.55^***^	−2.01^***^
History of convictions	Suspensions = yes: 1	–	–	−0.55^***^	–
	Other convictions = yes: 1	–	0.17^***^	–	−0.54^**^
History of crashes	Yes: 1	–	0.18^***^	0.67^***^	–
Gender	Male: 1	0.22^***^	–	1.16^**^	–
	older adults: 1	0.20^***^	0.20^**^	−0.92^***^	0.70^***^
Restraint system	Shoulder and lap belt not used: 1	0.19^***^	–	0.60^***^	–
Driver behavior	Under the influence of drugs or medication = yes: 1	−0.46^***^	–	−0.65^***^	–
	Failure to keep in proper lane = yes: 1	0.20^***^	0.36^***^	–	–
	Failure to yield right-of-way = yes: 1	0.76^**^	0.82^***^	–	–
Alpha					
	Intercept 2	−0.39^***^	−0.04^**^	0.37^***^	0.14^***^
	Intercept 3	−2.39^***^	−1.97^***^	−0.86^***^	−3.38^**^

^*^indicates values that are significant at the 0.1 level; ^**^indicates values that are significant at the 0.05 level; ^***^indicates values that are significant at the 0.01 level; bold numbers indicate values that are not significant at the 0.05 level; –indicates the default value.

**Table 5:** The marginal effects of truck occupant injury severity for different light conditions.

Variable	Overall sample	Daylight	Dark-not-lighted	Dark-lighted
	I(%) /II(%)/ III(%)	I(%) /II(%)/ III(%)	I(%) /II(%)/ III(%)	I(%) /II(%)/ III(%)
Front-to-rear = yes:1	−2.5/ 1.3/ 1.2	−1.9/ 1.0/ 0.9	−3.2/ 1.7/ 1.6	−1.0/ 0.6/ 0.4
Front-to-front = yes:1	−18.0/ 16.0/ 2.0	−18.1/ 14.7/ 3.4	−17.8/ 19.7/ −1.9	−17.9/ 10.3/ 7.6
Angle - front-to-side, right angle = yes:1	−6.0/ 7.7/ −1.7	−6.1/ 8.1/ −2.0	0.5/ 5.0/ −5.5	−9.6/ 5.5/ 4.1
Number of vehicles involved: Single vehicle = 1	−12.9/ −6.5/ 19.5	−29.3/ 1.4/ 27.9	−7.9/ −11.2/ 19.2	12.9/ −30.9/ 18
Rain, cloudy, snow, etc. = 1	−1.5/ 3.3/ −1.8	−0.2/ 3.4/ −3.1	−4.6/ 2.4/ 2.2	−0.4/ 0.2/ 0.2
Weekday = 1	−3.9/ 1.9/ 1.9	−2.9/ 1.5/ 1.4	−5.4/ 2.8/ 2.6	−3.1/ 1.8/ 1.3
Surface condition: wet, ice etc.: 1	3.6/ −1.8/ −1.8	5.3/ −2.7/ −2.6	4.1/ −2.1/ −2.0	−1.4/ 0.8/ 0.6
Roadway alignment: curve: 1	−5.2/ 1.4/ 3.8	−3.9/ 2.0/ 1.9	−7.5/ 3.9/ 3.6	−11.3/ 6.5/ 4.8
Roadway: grade etc.: 1	0.6/ −0.3/ −0.3	0.7/ −0.4/ −0.3	−4.8/ 2.5/ 2.3	1.0/ −0.6/ −0.4
Surface type Blacktop: 1	−0.2/ 0.1/ 0.1	0.5/ −0.3/ −0.2	−1.8/ −5.5/ 7.4	2.6/ 2.6/ −5.2
Speed limit ≤ 40 mile/h = 1	17.7/ −8.9/ −8.8	17.4/ −8.9/ −8.5	8.0/ −10.6/ 2.6	20.0/ −11.5/ −8.5
Speed limit 45–55 mile/h = 1	2.8/ −1.4/ −1.4	−0.4/ 0.2/ 0.2	6.4/ −3.3/ −3.1	14.1/ −3.8/ −10.3
Speed limit 60–70 mile/h = 1	−1.2/ 0.6/ 0.6	4.0/ −2.9/ −1.2	−1.1/ 0.6/ 0.5	11.9/ −6.8/ −5.1
Number of lanes per direction ≤ 2 lanes: 1	−1.4/ 0.7/ 0.7	−3.1/ 1.6/ 1.5	−5.2/ −0.9/ 6.1	0.9/ −0.5/ −0.4
Body type Truck-tractor: 1	3.0/ −1.5/ −1.5	0.7/ −0.5/ −0.2	1.5/ −0.8/ −0.7	1.3/ −4.1/ 2.8
Truck weight ≥33,001 lbs.: 1	9.6/ −4.8/ −4.8	12.8/ −8.9/ −3.9	10.1/ −2.2/ −7.9	8.3/ −10.0/ 1.7
Restraint system: Shoulder belt not used: 1	−25.4/ 10.9/ 14.5	−25.6/ 13.2/ 12.4	−23.5/ 6.4/ 17.2	−19.0/ 3.8/ 15.2
Vehicle age ≤ 5 years = 1	0.3/ −2.1/ 1.8	1.0/ −1.9/ 0.9	−4.3/ −3.4/ 7.7	3.7/ −12.6/ 8.9
Vehicle age>5 and ≤ 10 years = 1	0.2/ −0.1/ −0.1	1.0/ −0.5/ −0.5	−5.9/ 3.0/ 2.9	1.4/ −10.1/ 8.7
Gender Male = 1	5.3/ −4.8/ −0.5	3.5/ −2.5/ −1.0	5.9/ −14.2/ 8.3	−1.0/ 0.6/ 0.4
≤25 years = 1	3.1/ −1.6/ −1.5	2.0/ −1.0/ −1.0	−1.0/ 9.4/ −8.4	−2.6/ 1.5/ 1.1
≥65 years = 1	−6.1/ 3.1/ 3.0	−4.6/0.4/4.2	−4.8/ 2.5/ 2.3	1.4/ −5.9/ 4.5
License class compliance Valid = yes:1	3.6/ −1.8/ −1.8	2.2/ −1.6/ −0.6	7.6/ −10.2/ 2.6	5.5/ −2.9/ −2.6
Driver drinking Yes: 1	−13.9/ 7.0/ 6.9	−9.4/ 4.8/ 4.6	−7.8/ 4.0/ 3.8	−10.8/ 6.2/ 4.6
History of crashes Yes: 1	−1.3/ 0.65/ 0.65	−2.0/ −0.8/ 2.8	1.3/ −7.2/ 5.9	1.6/ −0.9/ −0.7
History of suspensions Yes: 1	−4.1/ 4.3/ −0.2	−2.5/ 1.3/ 1.2	−7.8/ 9.4/ −1.6	−3.4/ 1.9/ 1.5
History of speeding Yes: 1	2.5/ −1.2/ −1.3	3.2/ −1.6/ −1.6	1.1/ −3.1/ 2.0	−1.1/ 4.6/ −3.5
History of driving while intoxicated Yes: 1	4.7/ −2.4/ −2.3	7.0/ −3.6/ −3.4	9.6/ −5.6/ −4.0	0.4/ −0.2/ −0.2
History of other convictions Yes: 1	−0.2/ −1.1/ 1.3	1.0/ −2.2/ 1.2	0.6/ −0.3/ −0.3	2.9/ −1.6/ −1.3
Drowsy or fatigued Yes: 1	−37.4/ 18.7/ 18.7	−33.1/ 17.0/ 16.1	−51.4/ 26.6/ 24.8	−62.4/ 35.9/ 26.5
Under the influence of drugs or medication Yes: 1	−16.3/ 12.7/ 3.6	−10.2/ 5.2/ 5.0	−12.6/ 12.7/ −0.1	−0.7/ 0.4/ 0.3
Careless/inattentive Yes: 1	−18/ 9.0/ 9.0	−17.5/ 9.0/ 8.5	−27.8/ 14.3/ 13.5	−21.3/ 4.1/ 17.2
Failure to keep in proper lane Yes: 1	−21.6/ 8.9/ 12.7	−15.0/ 4.2/ 10.3	−18.0/ 5.2/ 12.8	−36.4/ 20.9/ 15.5
Failure to yield right-of-way Yes: 1	16.5/ −15.8/ −0.7	15.5/ −16/ −0.5	14.2/ −7.3/ −6.9	23.0/ −13.2/ −9.8

I indicates no injury; II indicates minor injury severity; III indicates major injury severity.

From the fitting results reported in Tables 4, 5, we first analyzed the corresponding explanatory variables under different light conditions individually, and then the similarities and differences among the different light conditions models were obtained via a comparative analysis. As can be observed from Table 4, each explanatory variable has one beta coefficient and one gamma coefficient if the variable violates the parallel line assumption. A variable is identified as a significant contributing factor if it has a *P-value* less than 0.05, which means that the estimated coefficient is significantly different from zero at the 95% confidence level. In addition, when the sign of the parameter is positive, the probability of a higher truck-occupant injury severity level is expected to increase when the corresponding value of the variable increases. As indicated in Table 5, the marginal effect not only indicates the changing trend of the probability of crash severity when the explanatory variable is modified but also tangibly indicates the change in the magnitude of the probability of the crash severity caused by a one-percent change in the explanatory variables while the other variables are held fixed.

### 5.1 Daylight conditions model

The estimation results of the daylight conditions model can be obtained from Tables 4, 5, and the corresponding significance tests and marginal effects of various explanatory variables are analyzed and explained below. Among the environment- and roadway-related variables, alignment (beta coefficient = 0.19, *P* < 0.05), weekday (0.14, *P* < 0.05), and the posted speed-limit category of 60–70 miles/h (0.15, *P* < 0.05) were positively associated with truck occupant injury severity. In addition, adverse road surface conditions (−0.26, *P* < 0.05) and the posted speed-limit category of less than 40 miles/h (−0.86, *P* < 0.05) were negatively associated with truck occupant injury severity. Similar results were also obtained from the overall sample model. Among these variables, adverse weather such as rain or snow during daylight decreased the probability of major injury severity (marginal effect = −3.1%). This may be related to more cautious driving behavior under adverse weather conditions during daylight; however, actual pre-crash operating speed was not available in the TIFA/FARS data. At the same time, the probabilities of minor injury severity (1.5%) and major injury severity (1.4%) increased during weekdays, which may be related to higher traffic volume during weekdays and an increased probability of severe truck-occupant injury outcomes. In addition, under poor road surface conditions such as wet, icy, or snowy roads, the probabilities of truck occupants suffering minor injury severity (−2.7%) and major injury severity (−2.6%) decreased. This result may be associated with more conservative driving behavior under adverse surface conditions, but it should not be interpreted as direct evidence of lower actual operating speed. In addition, curved roadway segments increased the probabilities of minor injury severity (2.0%) and major injury severity (1.9%). Curved roadway segments may involve more complex vehicle-control demands and are often associated with severe mechanisms such as rollovers, roadway departures, and severe collisions, which may aggravate truck occupant injury severity. The posted speed-limit categories also showed clear associations with injury severity: roadway segments with lower posted speed limits were associated with lower truck occupant injury severity, whereas roadway segments with higher posted speed limits were associated with higher injury severity. These results should be interpreted as associations between posted speed-limit environments and truck occupant injury severity, rather than causal effects of actual travel speed.

For the vehicle-related variables, body type (−0.23, *P* < 0.05) and vehicle weight (−0.63, *P* < 0.05) had significant negative impacts on occupant injury severity, whereas the restraint system not being used (1.27, *P* < 0.05) had a significant positive impact on occupant injury severity. Such results were consistent with those of the overall sample model. Among the variables, trucks weighing more than 33,001 pounds reduced the probability of major injury severity (−3.9%), whereas significantly increasing the probability of no injury (12.8%). To a certain extent, the truck-tractor body type reduced the probability of major injury severity (−0.2%). Large truck weights and a truck-tractor body type might protect truck occupants in collision, but it may aggravate the injury severity of other participants involved in the crash, so it is necessary to carefully analyze the impact of truck weight and body type from the overall crash level. For the restraint system, the probability of major injury severity (12.4%) increased significantly if the shoulder and lap belt were not used. Therefore, strengthening the truck configuration of the restraint system would play an important role in protecting truck occupants from serious crashes. In addition, a vehicle age of less than 10 years had a less significant negative impact on occupant injury severity. This might be caused by the better braking and safety performance in newer trucks, which can protect truck occupants to a certain extent.

For the driver-related variables, older adults (0.23, *P* < 0.05), drunk (0.47, *P* < 0.05), fatigued (1.64, *P* < 0.05), and drug-influenced (0.51, *P* < 0.05) drivers, in addition to careless driving (0.87, *P* < 0.05), and failure to stay in the proper lane (0.84, *P* < 0.05), all had significant positive effects on truck occupant injury severity. Among these, the probability of major injury severity caused by fatigue driving increased by 16.1%, which is the key contributing factor to severe occupant injury severity. Driving drunk (4.6%) or under the influence of drugs (5.0%) and failure to stay in the proper lane (10.3%) all caused the probability of major injury severity to increase significantly. In addition, older adults drivers increased the probability of major injury severity (4.2%), which is speculated to be related to their poorer health status and weaker resistance in collision. On the other hand, male drivers (−0.31, *P* < 0.05), those with a history of driving while intoxicated (−0.35, *P* < 0.05) and the failure to yield to the right-of-way (−0.76, *P* < 0.05) all had significant negative effects on truck occupant injury severity. Among them, male drivers (−3.5%) and drivers with a history of driving while intoxicated (−7.0%) reduced the probability of minor and major crashes, respectively. It is speculated that this may be caused by male drivers' tendency for having better driving skills and perceptions under daytime conditions. At the same time, drivers who have a history of drunk driving are more cautious, which may reduce the probability of major injury severity. Furthermore, when a truck fails to yield the right-of-way, it reduces the probability of minor injury by 16% while reducing the probability of major injury by 0.5%. Trucks have larger weights and longer lengths, and its safety is not guaranteed during lane changes or when it is not in the proper lanes. Therefore, it is important to ensure that trucks are in the proper lanes and do not switch lanes randomly.

For variables regarding crash characteristics, front-to-front (0.90, *P* < 0.05), angle-front-to-side (0.30, *P* < 0.05) and single-vehicle-involved crashes (1.45, *P* < 0.05) had significant positive effects on truck occupant injury severity. Among these variables, single-vehicle-involved crashes increased the probability of minor and major injury severity by 1.4% and 27.9%, respectively. When single-vehicle-involved crashes such as rollovers or collisions with fixed facilities occur, the injuries of truck occupants are often aggravated due to their higher center of gravity and greater inertia. In addition, compared with other collision manners, the probabilities of minor injury severity (14.7%) and major injury severity (3.4%) in front-to-front collision increases when the collisions immediately cause a larger inertia and impulse, which causes truck occupants to easily sustain more serious injuries.

### 5.2 Dark-not-lighted conditions model

For dark-not-lighted conditions model estimation results, significant tests were performed and marginal effects of explanatory variables were obtained, as can be observed in Tables 4, 5. Among the roadway and environment-related variables, weather conditions (0.23, *P* < 0.05), weekdays (0.27, *P* < 0.05), alignment (0.38, *P* < 0.05), grade (0.24, *P* < 0.05), and number of lanes per direction (0.26, *P* < 0.05) all had significant positive impacts on the occupant injury severity. On the other hand, adverse surface conditions (−0.20, *P* < 0.05) and the posted speed-limit category of 45–55 miles/h (−0.32, *P* < 0.05) were negatively associated with truck occupant injury severity. In addition, adverse weather conditions such as rain and snow caused the probabilities of minor and major injury severity to increase by 2.4% and 2.2%, respectively. It is speculated that in the absence of light at night, adverse weather has a greater impact on drivers' visual perception. The increased probability may be caused by drivers' more severely impaired vision, perceptions and operations in the absence of light at night. In addition, roadway segments with curves (3.6%) and grade (2.3%) both increased the probability of major injury severity. Curved or sloped roadway segments require higher truck operation performance and better controlling and braking systems. The lighting in these segments must be improved to reduce the driver's tension and vehicle operation requirements. For two-lane roadways per direction, the probability of major injury severity increased by 6.1%, which may be due to the relatively low design standard and fewer safety facilities on two-lane roadways. Similar to the daylight conditions, the probability of minor and major injury severity decreased by 2.1% and 2% under adverse surface conditions. This result may be related to more conservative driving behavior under wet or icy surface conditions. However, because actual pre-crash operating speed was not observed in the TIFA/FARS data, this explanation should be interpreted cautiously. Under dark-not-lighted conditions, roadway segments with lower posted speed-limit categories were associated with a reduced probability of major injury severity (−3.1%). Therefore, speed-management treatments, advisory-speed signs, reflective warning signs, and targeted enforcement may be considered as complementary safety measures to protect truck occupants from serious crashes at night.

For the vehicle-related variables, the truck weight (−0.51, *P* < 0.05) had a greater negative impact on the occupant injury severity, whereas the restraint system (1.18, *P* < 0.05) and vehicle age (0.22, *P* < 0.05) had greater positive impacts on the occupant injury severity. Among these variables, trucks weighing more than 33,001 pounds (−7.9%) reduced the probability of major injury severity, which may be because less damage is inflicted on the truck during a collision with the other participants such as passenger cars; moreover, the larger weight could protect truck occupants to a certain extent. In addition, not using shoulder and lap belts caused the probability of minor and major injury severity to increase by 6.4% and 17.2%, respectively. The proportion of truck occupants who do not wear shoulder and lap belts increases at night, which may be caused by truck occupants' poor safety awareness and less management efforts on rural roadways at night. Furthermore, vehicle age of less than 5 years (7.7%) and 5–10 years (2.9%) both increased the probability of major injury severity crashes. One possible explanation is that newer vehicles may be operated in different roadway or operational contexts, but the specific mechanism cannot be directly identified from the available TIFA/FARS variables. Therefore, the effect of vehicle age should be interpreted cautiously and examined further in future studies. However, some studies found that newer vehicles have better performance, which is conducive to mitigating crash severity. Therefore, it is necessary to analyze the specific mechanism of vehicle age in future studies.

For the driver-related variables, older adults drivers (0.24, *P* < 0.05), drunk driving (0.39, *P* < 0.05), fatigue driving (2.57, *P* < 0.05), driving under the influence of drugs (0.63, *P* < 0.05), careless driving (1.39, *P* < 0.05), and failure to stay in the proper lane (0.90, *P* < 0.05) all had significant positive impacts on occupant injury severity. Among these variables, risky and illegal driving behaviors such as fatigue (24.8%), distraction (13.5%), and failure to stay in the proper lane (12.8%) significantly increased the probability of major injury severity. In addition, male drivers (−0.29, *P* < 0.05), those with a history of other convictions (−0.03, *P* < 0.05), and failure to yield to the right-of-way (−0.71, *P* < 0.05) all had significant negative effects on occupant injury severity. Male drivers reduced the probability of minor injury severity by 14.2% but increased the probability of major injury severity by 8.3%. Therefore, heterogeneity among male truck drivers may exist, and more detailed research is needed in the future. When trucks failed to yield the right-of-way, the probability of minor and major injury severity were reduced by 7.3% and 6.9%, respectively. These results are generally consistent with the results for the daylight conditions.

For the crash-related variables, front-to-rear (0.16, *P* < 0.05), front-to-front (0.89, *P* < 0.05), and single-vehicle-involved crashes (0.40, *P* < 0.05) all had positive effects on truck occupant injury severity. Among these variables, in front-to-rear collisions, the probability of minor and major injury severity increased by 1.7% and 1.6%, respectively. In such cases, trucks are prone to lose balance, and secondary injuries such as those resulting from rollovers and collisions with fixed facilities aggravate the injury severity of truck occupants. In addition, front-to-front collisions caused the probability of minor injury severity to increase by 19.7%, but major injury severity decreased by 1.9%. In dark-not-lighted conditions, drivers are more likely to notice opposite cars from headlights, which may provide enough reaction time to avoid serious crashes. The occurrence of single-vehicle-involved crashes increased the probability of major injury severity by 19.2%, which may be caused by greater harm to truck occupants in serious crashes such as rollovers or rushing out of the roadway.

### 5.3 Dark-lighted conditions model

From the results of significance tests and the marginal effects of explanatory variables affecting truck occupant injury severity under dark-lighted conditions, as shown in Tables 4, 5, adverse weather (0.02, *P* < 0.05), weekdays (0.18, *P* < 0.05), and curve alignment (0.66, *P* < 0.05) were positively associated with truck occupant injury severity. Grade (−0.06, *P* < 0.05) and posted speed-limit categories were negatively associated with truck occupant injury severity. Among these variables, the probability of major injury severity increased on weekdays (1.3%), in adverse weather conditions such as rain and snow (0.2%), and on curved segments (4.8%). Compared with dark-not-lighted conditions, lighted conditions reduced the probability of major injury severity in adverse environments and roadway conditions to a certain extent, but they cannot completely prevent severe crash outcomes at night. In addition, for roadway segments with higher posted speed limits, the extent of probability changes for minor and major injury severity decreased accordingly (< 40 miles/h: −20%; 45–55 miles/h: −14.1%; and 60–70 miles/h: −11.9%). Therefore, lighting may help mitigate injury severity in some higher posted-speed-limit environments at night, although its effect is limited. Lighting improvement should be combined with speed-management treatments, reflective warning signs, advisory-speed measures, and targeted enforcement on high-risk rural roadway segments.

For vehicle-related variables, truck weight (−0.49, *P* < 0.05) had a negative impact on occupant injury severity, whereas restraint systems (1.11, *P* < 0.05) had a positive impact on occupant injury severity. Trucks weighing more than 33,001 pounds reduced the probability of minor injury severity by 10%, but the probabilities of no injury and major injury severity were increased by 8.3% and 1.7%, respectively. Although larger truck weights could protect truck occupants from minor injury severity, they aggravate occupant casualties to a certain extent. Therefore, heterogeneous truck weights might exist under lighted conditions at night, which requires more in-depth research. In addition, truck occupants not using shoulder and lap belts increased the probabilities of minor and major injury severity by 3.8% and 15.2%, respectively. Therefore, it is still urgent and essential to ensure the use of a restraint system for truck occupants in lighted conditions.

Among the driver-related variables, male drivers (0.06, *P* < 0.1) and young drivers (0.15, *P* < 0.1) were marginally positively associated with occupant injury severity, while driving while drunk (1.16, *P* < 0.05), fatigue (3.45, *P* < 0.01), distracted driving (0.24, *P* < 0.05), and failure to stay in the proper lane (2.13, *P* < 0.01) were significantly positively associated with occupant injury severity. The probability of major injury severity increased significantly when driving fatigue (26.5%), distracted (17.2%), or drunk (4.6%) or failing to stay in the proper lane (15.5%). Therefore, stronger nighttime safety management and targeted enforcement are needed to reduce risky and illegal driving behaviors among truck drivers. On the other hand, older adults drivers (−0.08, *P* < 0.05), valid license class compliance (−0.43, *P* < 0.05), or failure to yield to the right-of-way (−1.35, *P* < 0.05) had negative impacts on occupant injury severity. For older adults drivers, the probabilities of no injury and major injury severity increased by 1.4% and 4.5%, respectively, but the probability of minor injury severity was decreased by 5.5%. Additionally, heterogeneity among older adults drivers might exist, which requires further study. In addition, the driver's valid license class compliance (−2.6%) and failure to yield to the right-of-way (−9.8%) reduced the probability of major injury severity, which is consistent with the results of the overall sample model.

For the crash-related variables, front-to-front (1.05, *P* < 0.05) or angle-front-to-side (0.56, *P* < 0.05) collisions had positive impacts on truck occupant injury severity. Front-to-front and angle-front-to-side collisions increased the probability of major injury severity by 7.6% and 4.1%, respectively. These types of collisions may be related to reduced visual perception, shorter reaction time, and limited braking opportunities under nighttime lighted conditions, resulting in more serious collision outcomes. In addition, single-vehicle-involved crashes significantly reduced the probability of minor injury severity (−30.9%) but increased the probabilities of no injury (12.9%) and major injury severity (18%). Under the condition of light at night, the degree of truck occupant injury severity in single-vehicle-involved crashes had a certain heterogeneity, which needs further study. Although lighted conditions can increase the probability of no injury in single-vehicle-involved crashes, they increase the probability of major injury severity. There is a clearly complicated effect in lighted conditions regarding single-vehicle-involved crashes that requires further study.

### 5.4 Discussion

In this section, the contributing factors to truck occupant injury severity under different light conditions are explained in detail, accompanied by a comparative analysis and discussion of the similarities and differences of the explanatory variables in different light conditions, which facilitates our understanding of the effects of light conditions, as shown in Table 6.

**Table 6:** Comparative results of various exploratory variables in the three separate models.

Variable	Daylighted			Dark-not-lighted			Dark-lighted		
	I	II	III	I	II	III	I	II	III
Front-to-front	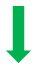	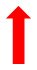	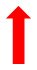	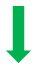	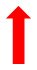	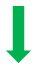	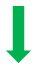	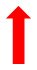	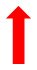
Single vehicle	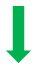 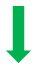 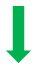	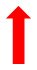	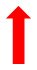 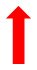 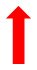	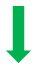	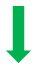 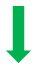	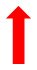 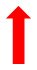	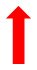 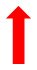	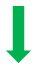 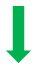 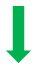	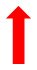 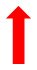
Rain, cloudy, snow, etc.	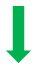	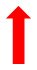	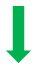	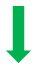	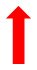	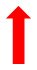	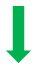	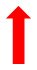	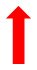
Roadway: grade, etc.	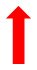	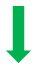	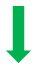	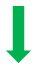	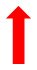	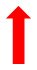	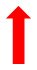	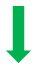	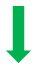
Posted speed limit 60–70 mile/h	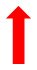	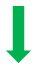	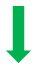	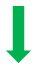	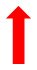	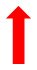	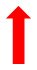 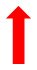	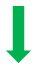	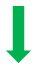
Number of lanes per direction ≤ 2 lanes	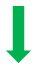	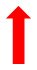	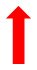	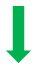	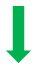	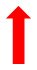	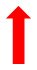	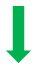	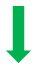
Male	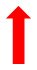	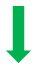	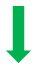	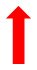	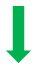 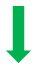	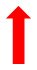	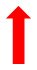	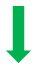	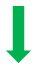
≤ 25 years	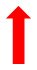	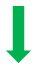	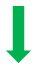	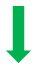	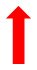	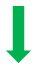	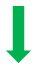	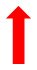	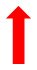
≥65 years	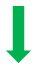	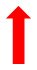	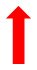	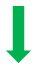	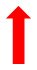	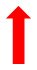	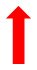	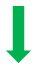	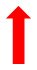
License class compliance Valid	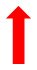	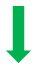	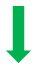	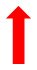 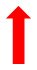	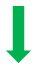 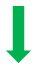	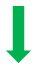	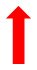	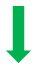	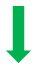
Driver drinking	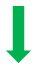	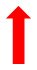	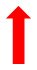	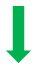	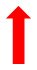	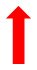	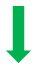	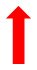	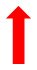
Drowsy or fatigued	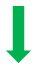 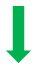 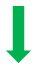	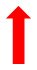 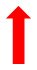	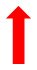 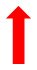	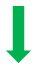 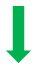 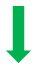	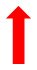 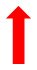 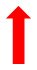	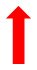 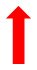 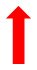	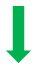 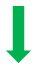 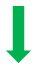	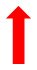 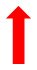 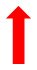	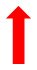 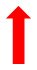 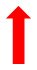
Under the influence of drugs or medication	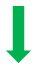 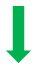	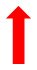	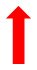	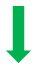 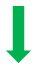	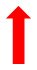 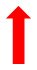	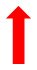	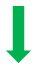	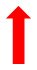	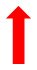
Careless/inattentive	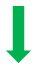 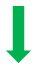	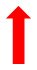	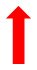	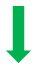 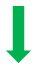 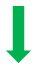	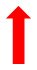 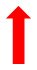	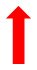 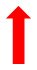	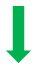 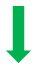 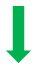	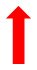	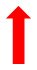 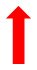
Failure to keep in proper lane	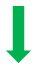 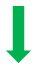	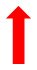	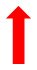 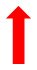	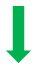 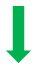	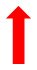	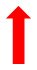 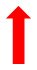	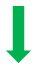 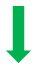 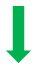	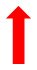 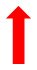	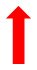 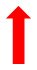
Yield right-of-way	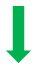 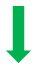	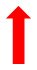 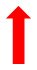	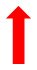	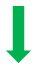	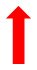	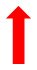	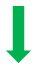 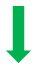 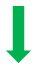	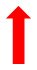 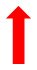	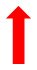

Arrow direction indicates the impact of exploratory variable on specific injury severity: 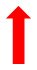 increase, 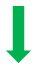 decrease. The number of arrows indicate the level of impact: one for less than 10%; two for more than 10% and less than 20%; three for more than 30%; I indicates no injury; II indicates minor injury severity; III indicates major injury severity.

The analysis of the driver-related variables showed that driver's invalid license class compliance; driving while drinking, fatigued, under the influence of the drugs, or distracted; failure to stay in the proper lane and failure to yield the right-of-way all increased the probability of serious injury severity under different light conditions, and similar results were also obtained by Helinä and Summala (3) and Wu et al. (23). Recent evidence from Islam (17) also indicates that fatigue-related large-truck crashes exhibit distinct injury-severity mechanisms compared with non-fatigue-related crashes, which supports the present finding that driver fatigue is a particularly important factor under nighttime lighting conditions. Moreover, it must be noted that the increased probability of major injury severity is more noticeable under dark-lighted or dark-not-lighted conditions, when drivers have more of a tendency to drive irregularly or illegally, which is speculated to be related to the weaker management intensity, driver's poorer mental state, obstructed vision, shorter sight distance, and slower response during nighttime. In addition, Anarkooli and Hosseinlou (12) also supported similar findings in their studies. Therefore, it is essential to ensure that truck drivers abide by transportation regulations in normal mental and physiological conditions. Particularly, under dark conditions, a driver's reasonable physiological status and appropriate operations can effectively mitigate truck occupant injury severity. Specifically, the regulation of reasonable driving hours and enough rest hours at night for truck drivers is recommended. Additionally, systems that monitor fatigued driving and driving under the drug or alcohol influence should be developed and installed in trucks, but Helinä and Summala (3) found that most of truck drivers expressed a negative view toward these technological countermeasure, and more suitable technological countermeasure should be developed in future. Furthermore, proper lanes and right-of-way for trucks should be planned to mitigate the injury severity of truck-involved crashes.

In addition, some driver-related contributing factors, such as young, older adults or male truck drivers, showed different effects on occupant injury severity in three separate models. Among these factors, when trucks were driven by young male drivers in daylight conditions, the probability of major injury severity was reduced, but the probability of minor and major injury severity were increased at night, which was more obvious under dark-not-lighted conditions. The performance differences of the same type of drivers under different situations were also indicated by Pahukula et al. (22) and Uddin and Huynh (1). Those authors speculated that this result may be related to a healthier body status, faster reaction and stronger resistance to collision among young male drivers, and such qualities are helpful in mitigating occupant injury severity during the daytime. However, at night, young male drivers may be relatively inexperienced and more prone to risky driving behaviors, which may increase the probability of severe injury outcomes. Especially under dark-not-lighted conditions, young male drivers may be more likely to engage in careless or risky driving behaviors, which may result in more serious casualties. This finding is consistent with Abdi and O'Hern (28), who found that young male truck drivers are over-represented in severe single-vehicle crashes at night, partly due to higher risk-taking behavior. Therefore, greater attention should be paid to the psychological and behavioral changes of young male truck drivers under nighttime conditions, and targeted management measures should be developed accordingly. Furthermore, the probability of major injury severity increased for older adults truck drivers under daylight and dark-not-lighted conditions, but this probability decreased significantly under dark-lighted conditions. Similar results were obtained by Behnood et al. (41) and Feng et al. (42), and we speculate that this may be caused by cautious driving habits and the abundant driving experience of older adults truck drivers at night. At the same time, older adults drivers tend to leave more space between vehicles and have sufficient reaction time to avoid serious crashes, so light conditions can effectively protect older adults truck drivers from major injury severity at night.

Regarding environment- and roadway-related variables, the results showed that the probability of major injury severity increased during weekdays and on curved roadway segments under different lighting conditions, whereas this probability was reduced under adverse surface conditions and on roadway segments with lower posted speed limits. Similar results were obtained in rural crash studies by Khorashadi et al. (5), Chen and Chen (6), and Xie et al. (7). It should be noted that the increased probability of major injury severity on curved roadway segments was more noticeable under dark-lighted conditions than under other lighting conditions. This may be because curved segments require greater visual perception, steering control, braking response, and vehicle stability management at night. Since actual pre-crash operating speed was not available, this result should not be interpreted as direct evidence that trucks were traveling at higher actual speeds. Instead, it should be understood as an association between curved roadway environments, posted speed-limit contexts, nighttime lighting conditions, and truck occupant injury severity. Roadway segments with lower posted speed limits were associated with a lower probability of major truck-occupant injury, whereas roadway segments with higher posted speed limits were associated with increased injury severity risk under certain lighting conditions. These findings reflect posted speed-limit environments rather than directly observed operating speeds. Therefore, safety management departments should combine nighttime lighting improvement with speed-management measures, advisory-speed signs, reflective warning signs, and targeted enforcement on high-risk rural roadway segments.

In addition, some contributing factors, such as adverse weather conditions, grade, roadway segments with higher posted speed limits, and two-lane roadway segments, had distinct effects on truck occupant injury severity. Pahukula et al. (22) and Uddin and Huynh (1) obtained similar results in their studies. Among these factors, the probabilities of minor and major injury severity increased under adverse weather conditions such as rain or snow under dark-not-lighted conditions, but these probabilities were reduced under daylight and dark-lighted conditions. This phenomenon may be related to more severely obstructed sight distance, poorer perception, and slower reaction under dark-not-lighted adverse-weather conditions. This interpretation is consistent with Al-Bdairi et al. (34), who found that large-truck run-off-road crash injury severity factors differed between lighted and dark conditions, and with Ullah et al. (32), who emphasized the joint influence of sight distance, road conditions, and weather on crash severity. Therefore, improved lighting may help mitigate occupant injury severity in adverse-weather scenarios on rural roadways, which was also supported by Lemp et al. (10) and Duvvuri et al. (55). At the same time, lighted conditions at night were associated with reduced probabilities of major injury severity on roadway segments with higher posted speed limits, grades, and two-lane configurations. Khorashadi et al. (5), Zhu and Srinivasan (11), and Chen et al. (56) obtained similar results in their studies. Additionally, roadway segments with higher posted speed limits were positively associated with increased probabilities of major injury severity, particularly under dark-not-lighted conditions. This may be because the absence of lighting further reduces drivers' visual perception and reaction opportunities in higher posted-speed-limit roadway environments. Lighting facilities in such segments may improve drivers' visual perception of roadway alignment and surrounding traffic conditions, thereby providing more time for appropriate vehicle operation. Therefore, it is recommended to prioritize lighting facilities, reflective warning signs, and speed-management treatments on rural roadway segments with grades, curves, two-lane configurations, and higher posted speed limits.

For the vehicle-related variables, contributing factors such as vehicle weight, body type, and restraint system in the three separate models had similar impacts on truck occupant injury severity. Among these variables, heavier and trailer trucks with the use of shoulder and lap belts decreased the probability of major injury severity under all lighted conditions. However, Zhu and Srinivasan (11), Lemp et al. (10) and Osman et al. (19) found that the occupant injury severity in crash level is significantly worse for heavier trucks and trailer trucks. Although large truck weights and a truck-tractor body type might protect truck occupants in collision, they may aggravate the injury severity of other participants involved in the crash, so it is necessary to carefully analyze the impact of truck weight and body type from the overall crash level. Moreover, it should be noted that trucks with trailers, larger weights and the use of a restraint system under daylight or dark-lighted conditions reduced the probability of major injury severity to a much lesser extent than under the dark-not-lighted condition. This may be related to more severe collision configurations or different roadway and operational contexts under lighted conditions, but the specific mechanism cannot be directly identified from the available TIFA/FARS variables. Therefore, the protective effects of vehicle-related characteristics should be interpreted cautiously. Therefore, it is essential to strengthen the safety design of trucks, such as vehicle body type, weight, and restraint system design, to ensure and improve truck safety performance.

The analysis of crash-related variables showed that front-to-rear collisions had similar impact on occupant injury severity in the three separate models. It should be noted that the probability of major injury severity caused by front-to-rear collisions was more greatly increased under dark-not-lighted conditions than under the other conditions, which may be caused by more severe obstructed sight and less reaction time to avoid rear-end collisions under such conditions. In addition, front-to-front collisions significantly increased the probability of major injury severity under daylight and dark-lighted conditions, but this probability decreased under dark-not-lighted conditions. This may be related to more cautious driving behavior and greater attention under darkness; however, actual pre-crash operating speed was not observed in the TIFA/FARS data. In addition, single-vehicle-involved crashes in both daylight and night significantly increased the probability of major injury severity, but the probability of minor injury severity is significantly reduced at night. When trucks roll over or collide with fixed objects in a single-vehicle-involved crash, serious occupant injury severity is often caused by the truck's larger weight, longer length, and higher center of gravity. However, truck drivers may behave more cautiously at night, which may partly explain the lower probability of major injury severity in some nighttime single-vehicle crashes. This interpretation should be treated cautiously because actual operating speed was not available. Therefore, vehicle manufacturers need to pay more attention to the safety design for single-vehicle-involved crashes, and some safety devices such as airbags should be installed to protect truck occupants from severe injury severity.

## 6 Conclusions and future directions

In present paper, contributing factors to truck occupant injury severity were analyzed under daylight, dark-not-lighted, and dark-lighted conditions on rural roadways. Truck-involved fatal crashes that mainly occurred on rural roadways from 2012 to 2016 were divided into three subsets according to the different light conditions, and the key factors from driver-, vehicle-, crash-, environment, and roadway-related variables contributing to truck occupant injury severity were explored individually in three separate models: rural daylight, rural dark-not-lighted, and rural dark-lighted conditions. Then, two types of likelihood ratio tests were adopted to verify that the three separate models were statistically justified. At the same time, the ordered logit model and generalized ordered logit model were used to fit the crash datasets in different light conditions. A comparison of the fitting parameters in different models revealed that the generalized ordered logit model showed better relative in-sample fit than the ordered logit model. Therefore, the estimation results from the generalized ordered logit model were adopted and analyzed in a subsequent study.

Then, key contributing factors to truck occupant injury severity were identified in three separate models, and a comparative analysis was performed to discuss the similarities and differences among the explanatory variables under different light conditions. Some explanatory variables had similar effects on occupant injury severity under the three light conditions, but other explanatory variables had distinct effects on occupant injury severity. Among these variables, driver-related variables, such as fatigue driving, drunk driving, driving under the influence of drugs and other irregular or illegal driving operations; vehicle-related variables, such as vehicle weight, body type and restraint system; environment- and road-related variables, such as adverse surface conditions and curved road segments; and crash-related variables, such as front-to-rear collisions, all had similar effects on occupant injury severity under the different light conditions. It should be noted that although such variables had similar effects on occupant injury severity under different light conditions, there were still magnitude differences among their marginal effects. Some recommended safety management strategies for different light conditions according to such differences were provided. On the other hand, contributing factors such as driver sex, driver age, weather condition, grade, roadway segments with higher posted speed limits, two-lane roadways, front-to-front collisions and single-vehicle-involved crashes had distinct effects on the occupant injury severity under different light conditions. Based on the discussion of differences among explanatory variables, several practical recommendations were provided for decision makers, safety managers, and vehicle manufacturers.

In addition, the results obtained from this study provide practical recommendations for safety management countermeasures on rural roadways under different light conditions, and some suggestions for planning or installing lighting facilities on rural roadways could also be given. Compared with the daylight conditions, the truck driver's fatigue, distracted and other irregular or illegal driving behaviors increased the probability of major injury severity to a greater extent at night, and this probability was increased significantly for young male truck drivers during nighttime. Therefore, truck drivers' physical status and behaviors should be checked carefully by safety management departments at night. More electronic law enforcement equipment could be installed on rural roadways, and its use could be maximized to reduce the frequency of irregular or illegal driving at night. Additionally, reasonable driving and resting hours should be regulated to ensure the proper mental and physical status of truck drivers. Moreover, the probabilities of minor and major injury severity were higher under adverse weather, on roadway segments with higher posted speed limits, and on two-lane roadway segments under dark-not-lighted conditions, whereas these probabilities decreased correspondingly under daylight and dark-lighted conditions. Therefore, installing or improving lighting facilities under adverse weather, on roadway segments with higher posted speed limits, and on two-lane roadway segments may help reduce truck occupant injury severity at night. At the same time, the probability of major injury severity associated with curved roadway segments and roadway segments with higher posted speed limits was higher under dark-lighted conditions than under other conditions. Therefore, lighting conditions alone cannot completely prevent severe truck-occupant injury outcomes, and additional safety management measures should be implemented under dark-lighted conditions. Specifically, reflective warning signs and speed-management treatments can be implemented on curved roadway segments and roadway segments with higher posted speed limits to improve nighttime truck safety on rural roadways. Finally, single-vehicle-involved crashes significantly increased the probability of major injury severity under all lighted conditions, but trucks with trailers, trucks with heavier weights and the use of a restraint system reduced the probability of major injury severity. Therefore, typical scenarios in a single-vehicle-involved crash, such as rollovers and collisions with fixed objects, should be simulated by truck manufacturers, and the safe design of truck weight, body type and restraint systems should be improved to protect occupants in such crashes.

Through analyzing truck-involved fatal crashes under different lighting conditions on rural roadways, this study provides useful and practical insights into truck occupant injury severity in severe crashes. Nevertheless, several limitations should be noted and addressed in future research. First, the selected explanatory variables were mainly constrained by the availability of information in the TIFA dataset. Some potentially important factors, such as passenger-car-related variables, pedestrian-related variables, detailed roadway geometry, traffic volume, enforcement intensity, driver physical condition, vehicle maintenance status, and crash dynamics, were not fully available and therefore could not be incorporated into the models. Future studies may use multisource crash data fusion to obtain more comprehensive and objective datasets. Second, the dark-lighted subset contained a relatively small number of observations compared with the daylight and dark-not-lighted subsets; therefore, the corresponding estimates should be interpreted as exploratory evidence rather than definitive conclusions. Third, this study was designed as an explanatory comparison of contributing factors across lighting conditions rather than a prediction-oriented modeling study. Thus, holdout validation, cross-validation, confusion matrices, and sensitivity/specificity measures were not incorporated in the original modeling framework. Future studies should further evaluate the predictive performance of light-condition-specific models using independent validation samples and classification-performance measures. Finally, as highlighted by recent temporal stability studies, the effects of certain contributing factors may change over time due to advances in vehicle safety technologies, roadway lighting systems, and safety management practices. Although the 2012–2016 TIFA data provide a useful baseline, future research should update the analysis with more recent data, especially post-2020 data, to assess temporal transferability and the stability of the identified contributing factors.

## Data Availability

The raw data supporting the conclusions of this article will be made available by the authors, without undue reservation.
